# CRITICAL ANALYSIS OF EXPERIMENTAL MODEL FOR STUDY OF ADHESIONS AFTER INCISIONAL HERNIAS INDUCED IN RATS’ AND REPAIR OF ABDOMINAL WALL WITH DIFFERENT BIOMATERIALS

**DOI:** 10.1590/S0102-67202015000300008

**Published:** 2015

**Authors:** Leonardo Carvalho SERIGIOLLE, Renato Lamounier BARBIERI, Helbert Minuncio Pereira GOMES, Daren Athiê Boy RODRIGUES, Sarah do Valle STUDART, Pedro Luiz Squilacci LEME

**Affiliations:** From the University Nove de Julho, São Paulo, SP, Brazil

**Keywords:** Experimental/surgery, Abdominal wall, Ventral hernia, Tissue adhesions/surgery, Surgical mesh/adverse effects

## Abstract

**Background:**

Adhesions induced by biomaterials experimentally implanted in the abdominal cavity are basically studied by primary repair of different abdominal wall defects or by the correction of incisional hernias previously performed with no precise definition of the most appropriate model.

**Aim:**

To describe the adhesions which occur after the development of incisional hernias, before the prosthesis implantation, in an experimental model to study the changes induced by different meshes.

**Methods:**

Incisional hernias were performed in 10 rats with hernia orifices of standardized dimensions, obtained by the median incision of the abdominal wall and eversion of the defect edges. Ten days after the procedure adhesions of abdominal structures were found when hernias were repaired with different meshes.

**Results:**

The results showed hernia sac well defined in all rats ten days after the initial procedure. Adhesions of the greater omentum occurred in five animals of which two also showed adhesions of small bowel loops besides the omentum, and another two showed liver adhesions as well as the greater omentum, numbers with statistical significance by Student's t test (p<0.05).

**Conclusion:**

Although it reproduces the real clinical situation, the choice of experimental model of incisional hernia repair previously induced implies important adhesions, with possible repercussions in the evaluation of the second operation, when different implants of synthetic materials are used.

## Abstract (pt)

**Racional:**

As aderências induzidas por biomateriais implantados experimentalmente na cavidade abdominal são estudadas basicamente com o reparo primário de diferentes defeitos produzidos na parede abdominal ou com a correção de hérnias incisionais realizadas previamente, sem definição precisa do modelo mais adequado.

**Objetivo:**

Descrever as aderências que ocorrem após o desenvolvimento de hérnias incisionais, antes do implante de próteses, em modelo experimental para estudo de alterações induzidas por diferentes biomateriais.

**Métodos:**

Foram realizadas hérnias incisionais em 10 ratos, com orifícios herniários de dimensões padronizadas, obtidos pela incisão mediana da parede abdominal e eversão das bordas do defeito. Após 10 dias foram evidenciadas aderências de estruturas abdominais quando as hérnias foram reparadas com diferentes próteses.

**Resultados:**

Foi possível evidenciar hérnias com saco herniário bem definido em todos os ratos já no décimo dia após o procedimento inicial. Aderências do omento maior ocorreram em cinco animais, dos quais dois também apresentaram aderências de alças do intestino delgado além do omento, e outros dois aderências do fígado juntamente com o omento maior, números com significância estatística pelo teste t de Student (p<0,05).

**Conclusão:**

A opção por modelo experimental de reparo de hérnia incisional previamente induzida, embora mimetize a situação clínica real, implica em aderências importantes, com possível repercussão na avaliação da segunda operação, quando são utilizados diferentes implantes de material sintético.

## INTRODUCTION

The intraperitoneal adhesions may be experimentally induced by different methods^2^ ^14^ ^20^ ^27^ ^28^. Biomaterial implants in the abdominal wall allowed the development of classical experimental models for the study of its biocompatibility. They can be basically divided on: 1) those which an incision is made with standard size on the linea alba and its repair is made with different prostheses in the first operation^11^ ^19^ ^21^; 2) on those that resect fragments with various formats of the wall^9^ ^22^ ^23^ ^30^ and also perform primary repair^1^ ^10^ ^29^; and on 3) those whose model is similar to the usual clinical condition, initially producing an incisional hernia, with its subsequent correction^4^. The focus of these studies is the consequence of the synthetic implants directly in contact with the abdominal viscera, a topic that has gained importance with the development of laparoscopy as an option for treating ventral hernias^5^ ^16^ ^18^ ^25^. From the experimental point of view, many authors make no distinction between such options, also calling incisional hernia when defects of abdominal wall are fixed in the first surgery^6^ ^25^ ^29^ ^30^.

The incisional hernia is a common complication after conventional abdominal operations, occurring mainly in smokers, obese or after surgical wound infection^5^ ^13^ ^26^ and its correction is usually performed with synthetic prostheses. The biomaterials used for this purpose must be inert, have good resistance and trigger small inflammatory response at the site where they have been implanted to be properly integrated to the tissues^22^ ^23^. When repaired with conventional technique, polypropylene is often used, due to easy handling and low cost, but this material can not stay in contact with the abdominal contents, because it may cause the formation of adhesions and the risk of an intestinal obstruction or enteric fistulae^6^ ^18^ ^25^. The compatible prostheses with the abdominal cavity have been developed primarily for use in laparoscopy^3^ ^16^, but they have high cost and, although minimizing, they do not prevent adhesions completely; therefore some other tactical options have been used to correct this condition. The voluminous incisional hernias also pose complex problems, of difficult resolution^5^, even with modern biomaterials available for this purpose, and the publication of several experimental studies on the subject shows that the employed materials still need improvement.

Although some authors do not adequately individualize the experiments that cause defects in the abdominal wall of animals, with primary correction, such models are clinically alike reconstructions of the abdominal wall for treating tumors or even performing flaps, as the rectus abdominis muscle flap transposition for breast reconstruction, when synthetic prostheses are also used, and the incisional hernia itself, which occurs some time after a surgical procedure. Both situations show different behaviors and improper definitions confuse distinct problems^6^ ^9^ ^19^ ^24^ ^25^ ^29^ ^30^.

The aim of this study is to demonstrate that the experimental models for assessment of adhesions, after incisional hernia repair previously induced, may be affected by the development of adhesions of various abdominal structures, even before the use of prostheses in reoperation.

## METHODS

**FIGURE 1:**
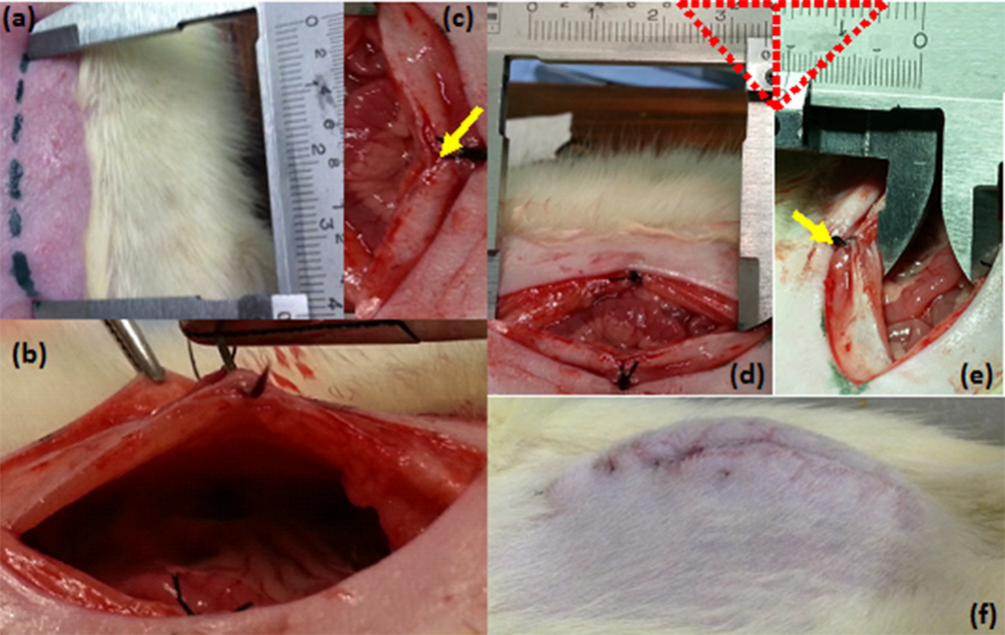
a) Midline incision of 4 cm; b and c) everted suture stitches not encompassing the peritoneum; d and e) defect of the abdominal wall with 3,5X1,5 cm; f) incisional hernia after 10 days of observation

This experiment was conducted at the University Nove de Julho, São Paulo, after approval by the Ethics Committee on Animal Use (AN 0034/13 protocol). The general rules for Experimental Research in the Advanced Surgical Skills Laboratory of the institution are strictly supervised and respect the current standards of "rational use of experimental animals"^17^. All animals received anesthesia before surgical procedures and before death, as well as the general care and standardized analgesia for postoperative period. Initially we present the results of the first 10 animals, from a study scheduled for 25 rats ( *Rattus norvegicus var. Albinus, Rodentia Mammalia* ) of the Wistar strain, distributed at random into groups of five animals each, which remained in individual cages before operations, with access water and standard food ad libitum, kept at a temperature of 25°C, light and dark cycles of 12 h. The animals weighed 339 g on average, were anesthetized by intraperitoneal injection of ketamine hydrochloride (50 mg/kg) and xylazine (10 mg/kg), a midline incision of 4 cm in length was perfomed, opening the abdominal cavity on the linea alba measuring 3,5 cm and a suture stitch was made in the middle third of each side of the incision, everting the edges of the rectus abdominis muscle, not encompassing the peritoneum, thus creating a defect with 3,5x1,5 cm. The experiment ended with a nylon 5-0 suture of the animals' skin, which were returned to the vivarium and observed for 10 days, waiting for the resultant incisional hernias (Figure 1).

On the 10^th^ day after the initial operation the rats were reoperated to evaluate the incisional hernias and adhesions formed initially. The values obtained in this first phase of the study were statistically analyzed with the Student t test.

To avoid conflicts of interest, donated synthetic prostheses were implanted in each animal after all adhesions were undone, cut in the shape of rhombus with 3,5x1,5 cm, corresponding to an area of 2,625 cm² and fixed with polyglactin 910 5-0 thread (Figure 5).

After more 10 days, the animals were re-operated for the removal of anterior abdominal walls in block, with any structures attached to the prosthesis, allowing macroscopic evaluation. These were fixed in 10% formol for 24 h and then in alcohol 70% to be routinely processed for histological and immunohistochemical study.

## RESULTS

**FIGURE 2:**
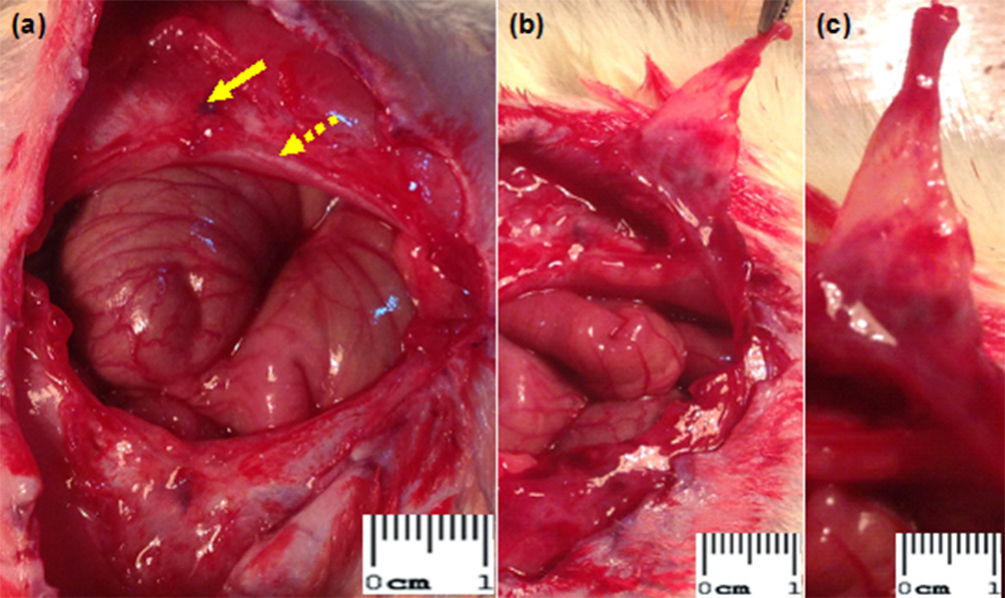
a) Appearance of the resulting herniary defect on the tenth day after surgery, everted stitch (arrow) and everted peritoneum (dashed arrow); b) and c) well-defined hernial sac, evidenced even with short time of observation

**FIGURE 3:**
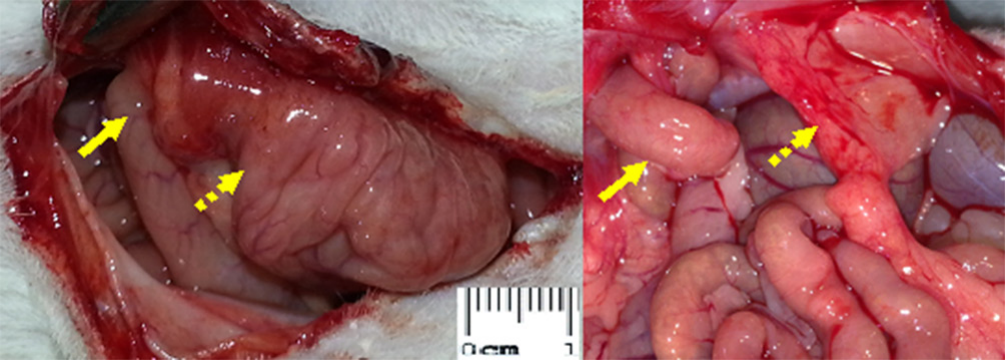
Adhesions to the small intestine (arrows) and the greater omentum (dashed arrows) wich occurred in two out of 10 animals (p<0,05)

**FIGURE 4:**
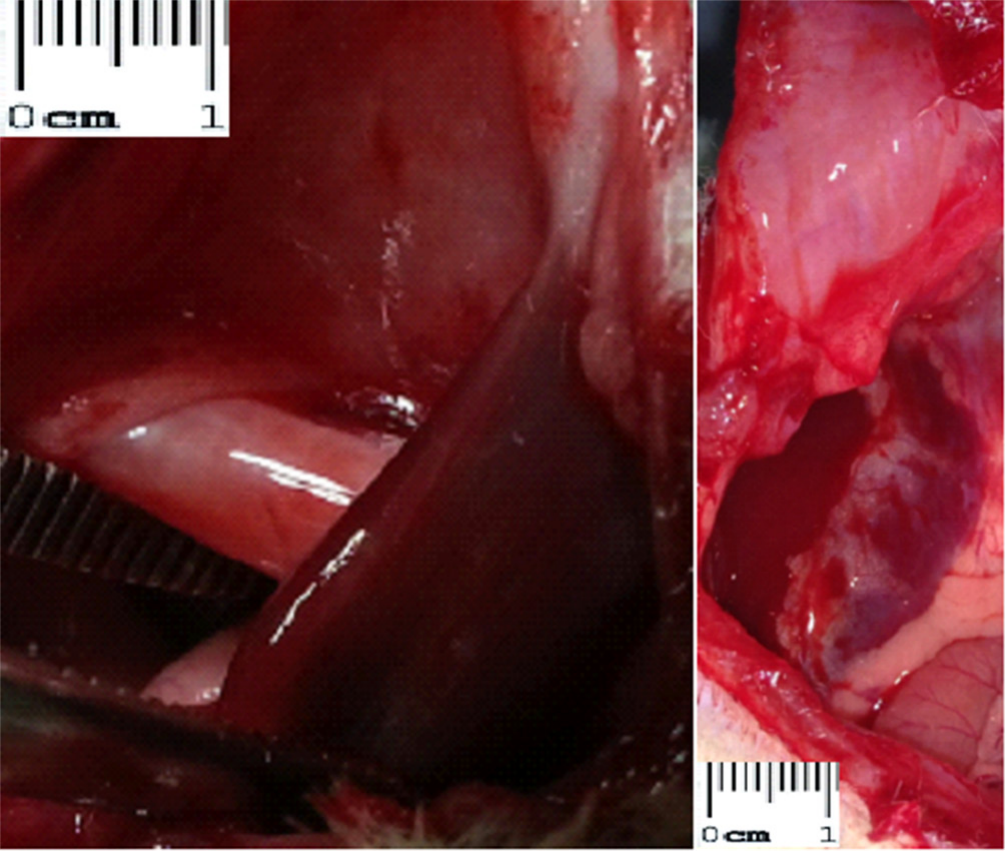
Adhesions to the liver that occurred in two out of 10 animals (p<0.05)

**FIGURE 5:**
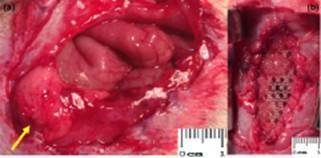
a) Omental adhesions (arrows) which occurred in 5 out of 10 animals studied (p <0.05); b) final aspect of the polypropylene prosthesis with 3,5x1,5 cm, which was sutured to the edges of the hernial orifice

All operated rats had incisional hernias (Figure 1f) with large orificiles and well defined hernia sacs already on the tenth day after the first operation (Figure 2). Five animals out of the 10 studied had dense adhesions of the greater omentum to the previously induced hernia, evidenced macroscopically (Figure 5). In the same five animals, two of them had the small intestine attached to the hernia orificile (Figure 3) and another two also presented, in addition to the greater omentum, adhesions to the liver, one of which had a large portion of the organ firmly attached to the hernia sac as well (Figure 4). Statistical analysis with Student's t test, confirmed statistical significance (p <0.05, Table 1).

**TABLE 1:** Adhesions and abdominal structures adhered to the hernia sac found 10 days after the completion of incisional hernias in 10 rats

Adhesions / total number of animals	10
No adhesions	5
Greater omentum (total number)	5	p <0,005
Greater omentum and small intestine	2	p <0,005
Greater omentum and liver	2	p <0,005
Greater omentum	1

## DISCUSSION

The abdominal wall hernias are frequent and of great clinical importance, justifying the inclusion of this theme in the line of research on cellular, molecular and tissue mechanisms of drugs' action and or non-pharmacological interventions on injury and repair, developed at the University Nove de Julho, in São Paulo. The study of hernias provides vast field of research on the cellular mechanisms, mutations and the ability to form collagen tissue, as well as on environmental factors and genetic alterations of this regulation, which are essential for proper postoperative healing. The progressive decrease of collagen with age, the changes of its ultrastructure, the further degradation and increased matrix proteases that accompany aging are also extensively studied, but several aspects not yet completely elucidated justify complementary research^13^ ^15^ ^26^. An important aspect of these studies is related to technologies involving the biophotonics, with laser employment (light amplification by stimulated emission of radiation), being an increasingly used option^30^. The development of this preliminary study is to evaluate the effects of the laser in the inflammatory response triggered by different biomaterials implanted in rat's abdominal wall defects, and the results of this initial observation led to a change in the planning of this research, in order to avoid the impact of the postoperative adhesions in the surgical procedure performed to create the incisional hernia.

Postoperative adhesions represent a complex problem after the manipulation of the abdominal cavity^2^ ^3^ ^10^ ^14^ ^28^ or situations that are accompanied by peritonitis^8^. Environmental factors such as smoking, factors that are inherent to the patient as obesity and genetics, as well as local complications of the incision, with wound infection, are important to the development of incisional hernias^13^ ^15^ ^26^. Published articles on the various options of synthetic prostheses used in the repair of such hernias^1^ ^3^ ^9^ ^18^ ^19^ ^22^ ^23^ ^29^ show that even the state-of-the-art materials, developed for direct contact with the abdominal cavity, despite decreasing, they can not completely prevent the formation of adhesions on these prostheses^4^ ^22^ ^23^ ^25^, demonstrating that these materials need to be improved. Several experimental studies describe tactics to minimize complications related to adhesions^2^ ^14^ ^20^ ^27^ and implanted biomaterials 3 6 16. Ingenious options as protection of the prosthesis with the greater omentum^6^, avoiding the adherence of the intestine or liver and even the previous implant on the wall, so that the synthetic material is enclosed by fibrous tissue^24^, and later used in the repair of defects, cannot always be applied in clinical practice.

The study of adhesions employs several experimental models: those which scarify viscera as the cecum, terminal ileum, sigmoid^20^ ^27^; perform fragment excision of the parietal peritoneum along with this scratching^2^ ^27^; evaluate effects of drugs such as piroxicam in the abdominal cavity^20^; of solutions with colloidosmotic action and absorbable polymer^27^; of phosphatidylcholine and plasminogen activator (protease)^14^; of different polypeptides^2^, and the application of barriers such as carboxymethylcellulose membranes^14^ ^25^ into the cavity. All of them represent examples where a defect is not created in the abdominal wall. 

Some researchers use the repair of defects produced in the abdominal wall to evaluate the biocompatibility of synthetic materials, performing the opening of the linea alba, biomaterial implant, fixation to the peritoneum and suture^11^ ^18^ or not of the muscles in different ways^1^ ^3^ ^16^ ^19^ ^24^. Another option would be to resect fragments of the abdominal wall in various shapes: rectangular 1 6 23, triangular^9^, squares^16^, ellipsoids^24^ ^30^, or even defects made laterally to the midline^9^ ^22^ ^28^. Regarding the repair of the defect, it can be made with all the fragments of the prosthesis within the cavity^3^ ^9^ ^6^ ^11^ ^19^ ^21^ ^30^ or just suturing the edges in a "bridge" shape^1^ ^6^ ^16^ ^22^ ^23^ ^24^, tactic used in this research.

Montes et al.^21^ employed in the same study two opening options of the abdominal cavity of the rat: by a median incision, polypropylene prostheses measuring 2x2 cm sutured with four stitches applied to the angles were implanted. In another group of animals, an incision in the form of "u" on the wall was performed, in order to deploy prostheses of the same material and size, but attached to the peritoneum with a fibrin sealant drop applied in its center, with no use of stitches. As two different approaches to open the cavity were used in the same experiment, the method used can be questioned, since the "u" incision, large enough to allow the placing of an implant of this size, produced greater damage to the wall than the one with the single midline incision. In the control group of these authors, when the "u" incision and repair without prothesis was performed, adhesion to the abdominal wall was observed in one out of five animals after 21 days and there was no adhesion in five median incisions, when prosthesis were not employed. In this study, when the abdominal closure with suture of the linea alba was performed, with no biomaterial implant (control group not presented in this publication), was found strong omental adhesion to the incision in one rat.

Baroncello et al.^9^ studied two different prostheses at the same time in 16 rabbits, performing triangular defects on each side of the anterior abdominal wall, lateral to the linea alba, fixing the prosthesis with polypropylene thread. This model is interesting since it compares simultaneously the different responses of the same animal to two different biomaterials, polyester with a collagen-polyethyleneglycol-glycerol foil and prosthesis made of extracellular matrix composed of swine intestinal submucosa, simplifying the statistical analysis.

Adhesion formation and the beginning of the healing process are precocious, as Vaz et al. demonstrated^30^, when they assessed their animals on the 1^st^, 2^nd^, 3^rd^, 7^th^, 20^th^ and 30^th^ days. van't Riet's et al. study^25^ also found early adhesions, which stabilized since the 7^th^ day of observation. Aydos et al.^7^, during the initial phase of their study in rabbits, only performed the opening of the linea alba, but modified the experimental model and opted for eversion of the edge of the incision because one of their animals had a strangulated hernia with necrosis of the cecum and enterocutaneous fistula. Claudio et al.^10^, studying a large number of animals for a longer period (90 rats - 45 days), reported nine deaths due to enteric fistulas and three due to intestinal obstruction following the implantation of different prostheses. Concerning this study, was lost one animal in the second phase, after implantation of the prosthesis, due to injury of intestinal loop by the suture, with fistula, peritonitis and death of the animal caused by technical fault. During the first phase of the study reported here, when the hernias were developed, there was no major complications.

Aydos et al.^7^ and Aramayo et al.^4^ performed continuous sutures everting the edges of the opening of the abdominal cavity in rabbits, sectioning^4^ or not^7^the angles of the incision to create the herniary orificile. We have gotten the same result with only a stitch in the middle third of each side of the midline incision. The choice for a few stitches and not to encompass the peritoneum was made to reduce handling and minimize surgical trauma; although the resulting hernias were satisfactory for the study, this maneuver failed to reduce the incidence of adhesions near the hernia orificile. Aydos et al.^7^ reported the occurrence of adhesions in five out of 15 rabbits 30 days after the first operation. Two animals presented adhesions of the cecum and ascending colon and another three omentum adhesions, all of them undone when laparoscopic surgery was performed. They described loose adhesions, undone with no difficulty in four animals, but in one of them it was necessary the use of scissors and after 60 days adhesions of the cecum were revealed in only two of those 15 rabbits. Aramayo et al.^4^ repaired the hernia orifices also after 30 days, but did not describe adhesions resulting from the first operation. They corrected the incisional hernias with three different prostheses measuring 7x5 cm and performed the repair in the fourth group as well, with 10 animals in the whole, using conventional technique without prosthesis (bilateral longitudinal peritoneum-aponeurotic transposition), a tactic that uses the hernia sac, reaching better results in this group (absence of adhesions). Considering that the most appropriate prosthesis for implantation in contact with the abdominal organs used in this study (low-density polypropylene, polydioxanone and oxidized regenerated cellulose) did not cause adhesions just in two of 10 animals (20%), it was confirmed the superiority of the autologous tissues of the herniary sac itself and the need to improve the biomaterials available. In this study, significant adhesions were found in half of the studied rats, proving to be more suitable for evaluation of adhesions the implantation of the synthetic material right in the first surgery, when the defect in the abdominal wall is performed.

Focused on the prevention of adhesions with different liquid barriers that can be applied during laparoscopy (hyaluronic acid and icodextrin solution for use in dialysis), van't Riet et al.^25^ reported the occurrence of loose adhesions of the omentum on polypropylene prostheses in all rats studied already on the seventh day. Although the animals here studied showed significant signs of inflammatory activity 10 days after the first operation, with edema and increased bleeding during handling and developed well individualized hernia sacs, they presented firm adhesions in this short observation period, even before the biomaterial implant.

There is a certain confusion related to the terminology used in the studies concerning defect repair in the abdominal wall of experimental animals. The most appropriate way would be the expression "defect correction"^1^ ^8^ ^18^ ^23^ ^28^ rather than "incisional hernias correction". Aramayo et al.^4^ used the correct denomination and although Aydos et al.^7^ have also produced incisional hernias experimentally, their aim was to perform videolaparoscopy surgery, not to correct the condition. Some authors accurately used the term "peritoneostomy"^10^ ^12^, a possible option, but a large number of articles incorrectly describe "incisional hernia" for situations in which the opening of the cavity or the resection of a fragment of the wall, with various shapes and sizes, is primarily corrected^6^ ^9^ ^19^ ^24^ ^25^ ^29^ ^30^.

## CONCLUSION

Experimental models that perform simple opening or resection of a segment of the abdominal wall, with immediate repair using different biomaterials, are more suitable for the study of postoperative adhesions. The prior development of incisional hernia, although similar to the clinical condition, resulted in significant adhesions, with possible repercussions in the final evaluation, after the second operation performed to repair the hernia orificile.
